# High crystallinity and polar-phase content in electrospun P(VDF-TrFE) nanofibers with low molecular weight

**DOI:** 10.1063/5.0267697

**Published:** 2025-05-16

**Authors:** Wenyi Zhu, Guanchun Rui, Yongsheng Chen, Bo Li, Shihai Zhang, Patrick T. Mather, Q. M. Zhang

**Affiliations:** 1 School of Electrical Engineering and Computer Science, Materials Research Institute, The Pennsylvania State University, University Park, Pennsylvania 16802, USA; 2 Arkema Inc., 900 First Avenue, King of Prussia, Pennsylvania 19406, USA; 3 PolyK Technologies, State College, Pennsylvania 16801, USA; 4 Department of Chemical Engineering, The Pennsylvania State University, University Park, Pennsylvania 16802, USA; 5 Department of Materials Science and Engineering, The Pennsylvania State University, University Park, Pennsylvania 16802, USA

## Abstract

Electrospun piezoelectric nanofibers from polyvinylidene fluoride (PVDF) have been widely used in many applications. In PVDF-based polymers, the molecular weight (M_w_) plays an important role in determining both crystallization and polarization responses. In the past, polyvinylidene fluoride trifluoroethylene [P(VDF-TrFE)] electrospun nanofibers were produced strictly from high molecular weight polymers (M_w_ > 200 kDa). Here, we study the electrospun P(VDF-TrFE) nanofibers from comparatively lower M_w_ polymers (M_w_ ∼ 100 kDa). We demonstrated a highly electroactive phase in electrospun P(VDF-TrFE) nanofibers without post treatments. During electrospinning, shorter P(VDF-TrFE) polymer chains exhibited higher mobility, which facilitate the formation of all-trans ferroelectric crystals with high crystallinity. By optimizing the mean size of electrospun nanofiber through tailoring the solution concentration and other controlling parameters, P(VDF-TrFE) nanofibers achieved the crystallinity as high as 67% and all-trans conformation reached 79%. The results pave a way for improving the electroactive performance in ferroelectric polymer electrospun nanofibers.

## I. INTRODUCTION

Piezoelectric polymers have found widespread applications in wearable sensors, actuators, transducers, energy harvesting, and biomedical devices due to their light weight, pliability, low process cost, and biocompatibility in recent decades.^1,2^ Piezoelectric polymer nanofibers are unique materials that combine the piezoelectricity and nanoscale engineering to reach high performance based on their high aspect ratio and enhanced surface area of nanofibers.^3–8^

Electrospinning is a well-established, straightforward technique to fabricate continuous polymer nanofibers with 1D nanostructure of sub-micrometer diameter, offering significant versatility for various applications. Customized electrospinning setups and mechanisms have been described extensively in the literature.^9,10^ During the electrospinning process, a polymer solution is precisely dispensed by a syringe pump through a fine, electrified needle, forming a Taylor cone at the tip due to the applied high voltage between the needle tip and receiver that overcomes surface tension, which subsequently produces polymer nanofibers. Because the nanofibers are subjected to elongation due to the strain along the direction of the electric field, the ferroelectric nanofibers may be automatically poled a under high electric field during the electrospinning process. Over the past decades, this method has been widely used to fabricate piezoelectric polymer nanofibers for a broad range of applications.^11–14^ Many studies have been conducted to optimize electrospinning parameters to enhance the functional performance of piezoelectric nanofibers. For example, a higher rotation speed of a spinning drum collector can increase the mechanical strains on nanofibers and increased anisotropy and uniformity of the fibers.^15^ It has been shown that the piezoelectric properties of these nanofibers can be tailored by controlling electrospinning parameters, such as polymer solution concentration, solvent choice, flow rate, drum–collector rotation speed, temperature, humidity, the distance, and high voltage between the needle tip and the collector.

Of particular interest in the electrospinning of polymers with an unknown level of entanglements in solutions is the required concentration for adequate fiber formation. Nasouri *et al.* investigated the role of polymer concentration, viscosity, and Berry number for a model system [polyacrylonitrile (PAN) in N,N-dimethylformamide (DMF)], demonstrating that the Berry number (*Be* = [*η*]C) effectively predicts fiber formation and morphology.^16^ They found that uniform nanofibers formed within 3.5 < Be < 7.5, while solutions with Be < 2 produced droplets due to insufficient chain overlap, and Be > 7.5 resulted in excessive viscosity. Empirical scaling relationships were established to relate viscosity, concentration, and fiber diameter, serving practical utility. Their findings reinforce the Berry number as a key parameter in electrospinning, and we use that in the present study.

Polyvinylidene fluoride (PVDF)-based ferroelectric polymers are most widely used for electrospun piezoelectric nanofibers, because of their high polarizations and piezoelectric responses, among the known polymers.^17^ In polymers, molecular weight plays an important role in determining how the polymer behaves under these processing conditions, impacting both the morphology and crystalline structure.^18^ In the literature, the M_w_ of the polyvinylidene fluoride trifluoroethylene [P(VDF-TrFE)] for electrospun nanofibers was usually greater than 200 kDa.^3,4^ This is due to the fast propagation process during the free-radical polymerization with less controllability. The use of such molecular weights results in a narrow spinnable window (10–20 wt. %) for P(VDF-TrFE) solution.^6^ Moreover, the long polymer chains may contain more structural defects (branches, regiodefects, and tacticity defects) that further affect the formation of the crystals and ferroelectric domains.^19–21^ On the other hand, semicrystalline polymers with low molecular weight and shorter chains may have better chain mobilities to facilitate crystal growth under an electric field and, thus, improve the crystallinity and polar conformations in the electrospun fibers. Consequently, lower molecular weight can lead to better polarization and piezoelectricity. For example, Zahari *et al.* studied the M_w_ effect of P(VDF-TrFE) 70/30 copolymers, in M_w_ ranging from 180 to 1000 kDa, on electrospun nanofibers. They observed that the fraction of the β phase (polar phase) in electrospun nanofibers as well as the piezoelectric response increased with reduced M_w_.^22^ The results seem to be consistent with what was observed in normal P(VDF-TrFE) polymer films.^23^

Here, we study the webs of electrospun P(VDF-TrFE) (70/30 mol. %) nanofibers with low molecular weight (M_w_ = 100 kDa). We found that while the processing conditions for producing electrospun nanofibers of the low M_w_ copolymer were quite different from those used for high M_w_ copolymers (see Table S1 in the supplementary material), a favorable window could be established. Importantly, the electrospun nanofibers produced high crystallinity (67%) and polar-phase content (79%), higher than those from high M_w_ (>200 kDa) polymers.^3,4^

## II. EXPERIMENTAL SECTION

### A. P(VDF-TrFE) solution preparation

The P(VDF-TrFE) copolymer with monomer molar ratio VDF/TrFE 70/30, Arkema, France) was provided by PolyK Technologies. The melt flow index (MFI) is 15 g/10 min at 230 °C/5 kg and the weight-average molecular weight (M_w_) is 100 kDa, as reported by the vendor. Prior to electrospinning, P(VDF-TrFE) with prescribed mass was dissolved in a mixed solvent system (DMF/acetone) with the volume ratio of 5:4 at room temperature overnight. The viscosity of solution with concentration less than 18 w/v% was measured by a microVISC portable viscosity meter (RheoSense) with a sensor labeled 23HA05100661 in the auto mode.

### B. P(VDF-TrFE) electrospun nanofiber preparation

P(VDF-TrFE) nanofibers were prepared by using a commercial electrospinning equipment (Fluidnatek LE-50 by Bioinicia, Spain). During the electrospinning process, the solution concentrations, needle size, flow rate, receiver distance, and applied voltage between the needle tip and collector were carefully controlled (see the discussion Sec. III). The electrospun P(VDF-TrFE) nanofiber mat was collected on an aluminum foil wrapped roller collector (diameter: 10 cm and length: 20 cm). The fiber mat on the aluminum foil was removed from the roller collector and baked in a vacuum oven at 60 °C to remove the solvent residue.

### C. Microstructure characterization

The morphology of P(VDF-TrFE) nanofibers was characterized by a scanning electron microscope (SEM, Verios G4, Thermo Fisher Scientific). The fiber dimension distribution was analyzed using ImageJ software (NIH), with which 300 individual diameter measurements were conducted for each SEM image.

### D. Structural characterization

XRD (Malvern Panalytical Empyrean III), FTIR (PerkinElmer Spectrum Two), and DSC (TA Instruments DSC Q2000) were used to characterize the structural changes from the electrospun P(VDF-TrFE) nanofibers prepared with abovementioned different electrospinning parameters. The heating and cooling rate was 10 °C/min in DSC measurements.

## III. RESULTS AND DISCUSSION

We note that the solution concentration for most electrospun P(VDF-TrFE) nanofibers in the past (for high M_w_, >180 000 g/mol.) was in the range between 10 and 20 wt. %. However, we could not produce electrospun fibers using solutions of the low M_w_ PVDF polymers in this concentration range.

Both the concentration and viscosity of the polymer solution are important in enabling electrospinning and defining the final morphology of nanofibers. The Berry number, a product of intrinsic viscosity and concentration, was used as the process index in the electrospinning process to control the diameter of nanofibers.^16^ In general, to produce electrospun nanofibers requires the solution with Berry numbers above 3, which we will show to be the case here. In this study, a series of solutions of P(VDF-TrFE) (70/30) with concentration at 8, 10, 12, 14, 16, and 18 w/v% (g/dl) were prepared in mixed solvent DMF/acetone (5/4, volume ratio) and their viscosity was characterized. The results are presented in Fig. S1(a) in the supplementary material. From the relationship of concentration with the viscosity of diluted P(VDF-TrFE) solution, the specific viscosity can be obtained as described in the literature.^24^ In Fig. S1(b) in the supplementary material, we extrapolated the curve of reduced viscosity *η*_sp_/*c* vs solution concentration *c* and obtained the intrinsic viscosity [*η*] of 0.126 dl/g.

To guide the electrospun nanofibers of the low M_w_ PVDF polymers, we calculated the Berry numbers of the P(VDF-TrFE) solutions with different concentrations. As shown in Fig. S3(c) in the supplementary material, the solutions with concentrations higher than 22 w/v% have the Berry number of 2.77, from which could be noted as spinnable compositions. Therefore, we conducted electrospinning with the concentrations from 22 to 36 w/v%. The detailed electrospinning parameters in this study are summarized in Table I.

**TABLE I.** Experimental parameters for the electrospinning process of nanofibers.

Electrospinning Parameter	Value
Needle size	20 gauge (ID: 0.603 mm)
Needle raster distance and speed	2 cm and 50 mm/s
Tip–collector distance	10 cm
Voltage at the needle tip	12 kV
Voltage at the receiver	−1.1 kV
Drum rotation speed	250 rpm
Flow rate	1 ml/h

### A. Morphology study of P(VDF-TrFE) nanofiber mats

**FIG. 1.**
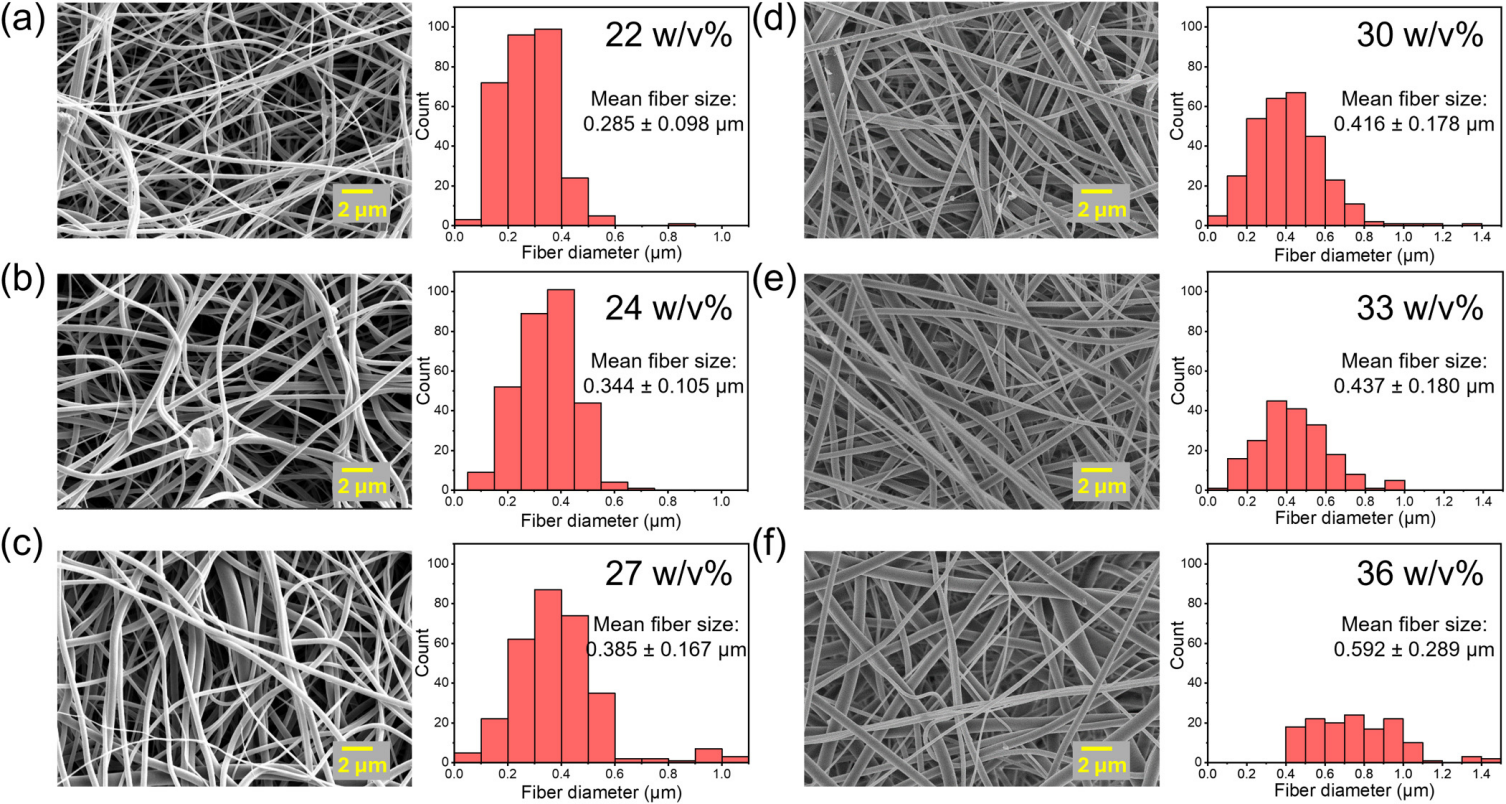
SEM images and fiber size distribution from P(VDF-TrFE) (70/30) electrospun fibers from solution with concentration varied from 22 to 36 w/v%: (a) 22, (b) 24, (c) 27, (d) 30, (e) 33, and (f) 36 w/v%.

**FIG. 2.**
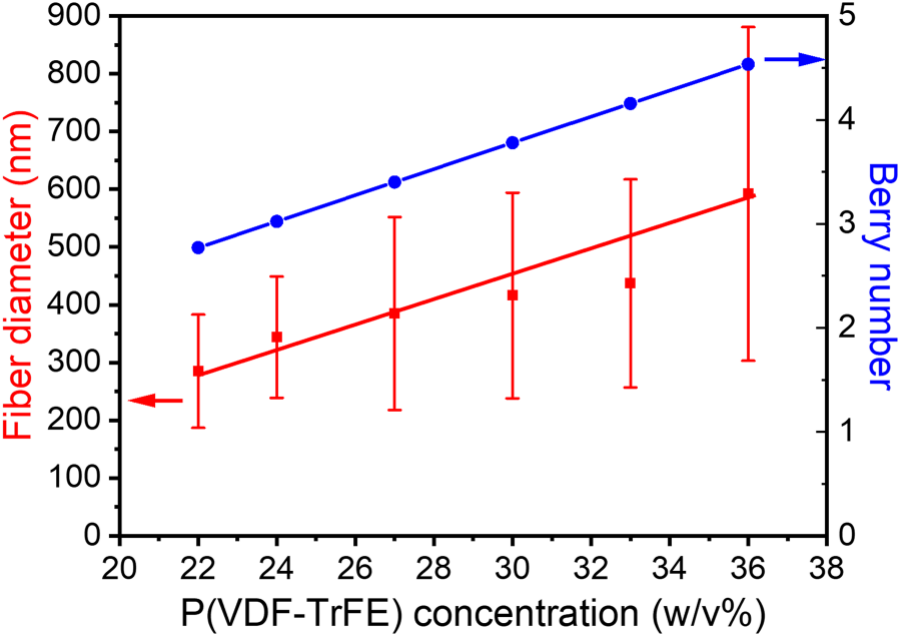
The mean diameter of P(VDF-TrFE) nanofibers and Berry number (*Be*) vs the solution concentration. Data points are shown, and solid lines are drawn to guide eyes.

SEM was carried out to probe the morphology of electrospun nanofibers with different concentrations. Furthermore, the fiber dimensions and distributions were also calculated based on the SEM image, as shown in Fig. 1. The results indicated that the mean fiber diameter increased from 285 to 592 nm as the solution concentration increased from 22 to 36 w/v%.^25^ The relationship of the Berry number, *Be*, with the concentration was presented in Fig. 2, showing that *Be* also increases with concentration, as expected.

### B. Structural study of P(VDF-TrFE) nanofibers

**FIG. 3.**
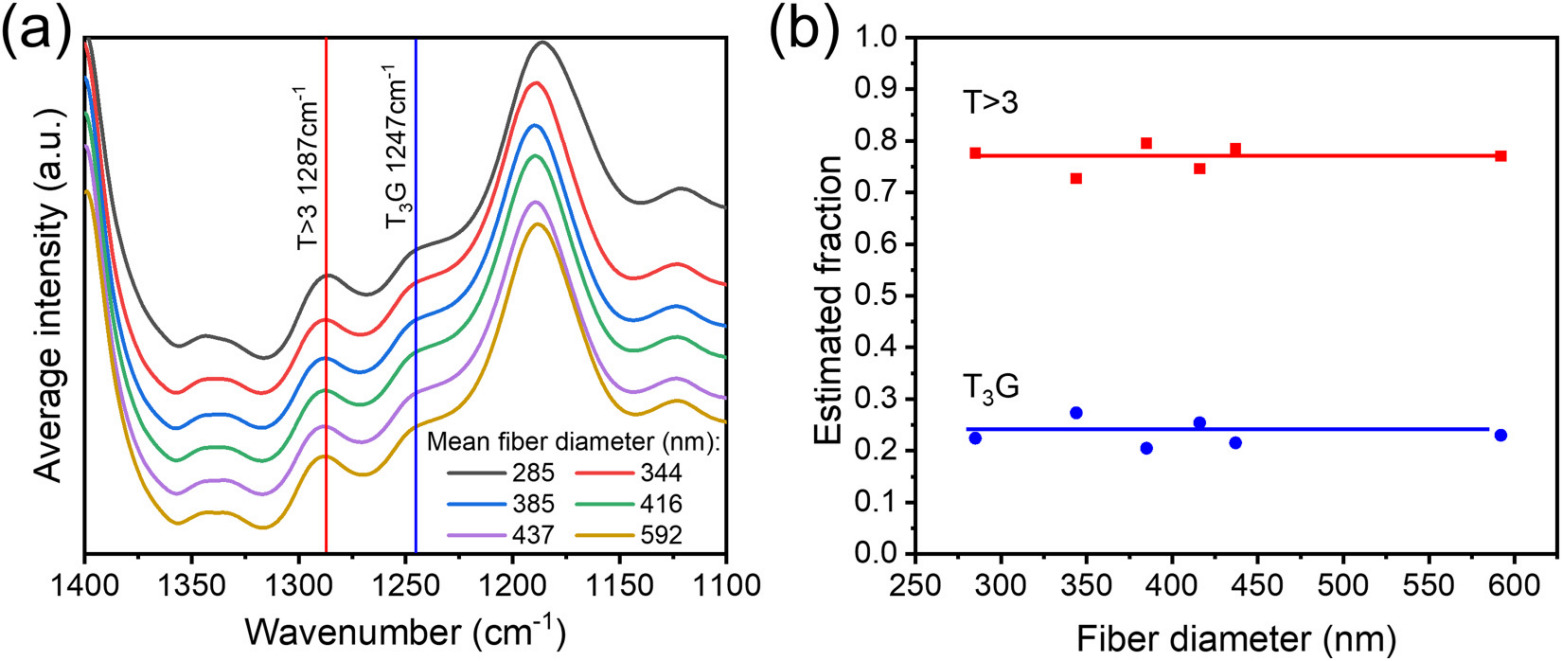
(a) FTIR spectra of P(VDF-TrFE) electrospun nanofibers and (b) β(1287 cm^−1^) and γ phase (1247 cm^−1^) fractions. Data points are shown and solid lines are drawn to guide eyes.

A series of structural characterizations were conducted on the full set of electrospun nanofibrous webs. The FTIR spectra were used to characterize the molecular conformations in the electrospun fibers, and the results are presented in Fig. 3(a). The data revealed that β (T > 3) and γ (T_3_G T_3_G′) conformation were dominant, as revealed by Li *et al.*^26^ To determine the exact content of different conformation contents, the peak to valley height ratio (P2VHR) method was used.^26^ We here used the exclusive T > 3 conformation at 1287 cm^−1^ and exclusive T_3_G T_3_G′ at 1247 cm^−1^, as followed by Ref. 27. As shown in Fig. 3(b), the T > 3 β phase at 1287 cm^−1^ was present between 72% and 79% samples over the full range of mean diameter, while the γ phase indicated by the 1247 cm^−1^ absorbance was present between 28% and 21% for the same. This evidence indicates the stable ferroelectric phase with high content of polar conformation exists in all the electrospun P(VDF-TrFE) fiber mat with mean diameters ranging from 250 to 600 nm, regardless of their diameter and distribution differences.

**FIG. 4.**
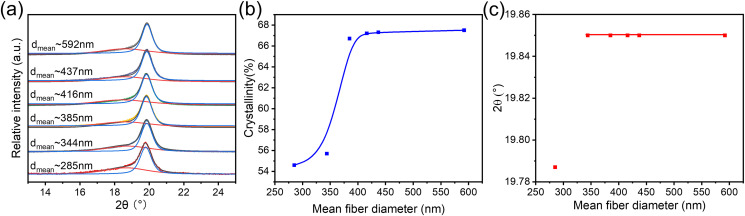
(a) Peak deconvolution of the XRD results for P(VDF-TrFE) nanofibers with different mean diameter; (b) estimated crystallinity from XRD fitting results; (c) (110/200)_β_ peak position vs fiber diameter. Data points are shown, and solid lines are drawn to guide eyes.

XRD was performed to collect the crystallography information of the P(VDF-TrFE) electrospun nanofibers. The multipeak fitting method was used for the deconvolution of the XRD peaks and to quantify the crystallinity in the P(VDF-TrFE) electrospun nanofiber [Fig. 4(a)]. As shown in Fig. 4(b), the crystallinity (X_c_) increased with the increase in the mean fiber diameter until it reached a plateau for the fiber with a mean diameter of 416 nm (for which X_c_ = 0.672). Beyond this mean diameter, X_c_ remains almost unchanged. Moreover, the 2θ angle position of the (110/200)_β_ plane was at lower position (larger *d*-spacing) for fibrous webs featuring the smallest mean diameter from low concentration solution (22 w/v%), beyond which this value increased (*d*-spacing decreasing) and stayed almost unchanged at the same higher position for nanofibers with larger mean diameter from concentration greater than 22 w/v% as shown in Fig. 4(c). This is in line with the observation of in our recent studies, in which the more stable typical ferroelectric phase appears at a higher 2θ angle than an unstable one.^1,28^ Based on these observations, we suspect that during the electrospinning process, a lamination crystallization process happened during the fiber solidification (i.e., the fiber sprayed out from the Taylor cone and dried by the surrounding air). Thicker fibers will dry more slowly, providing mobility for a longer time that promotes the formation and growth of better crystals. By comparison, lower concentration solutions yield thinner fibers that dry more quickly, resulting in the kinetic “trapping” of crystals that are defective and incompletely developed.

**FIG. 5.**
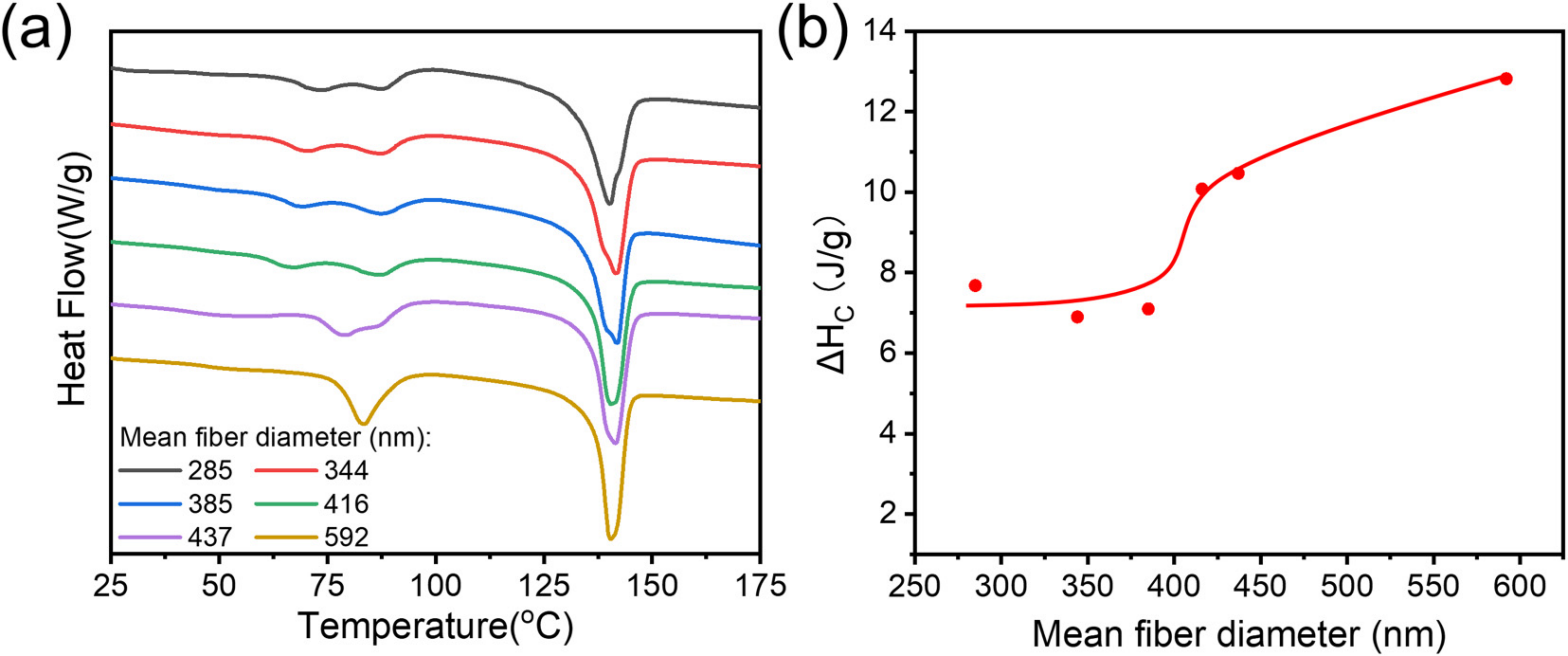
(a) DSC curves for P(VDF-TrFE) nanofibers with different mean diameters; (b) calculated ΔH_C_ with the increase in the fiber diameter.

DSC was conducted to further explore the structural change in electrospun P(VDF-TrFE) (70/30) nanofibers with different mean fiber diameters. The results of first heating run are shown in Fig. 5(a). There was only one primary melting temperature at near 141 °C, and no significant change with the change in the fiber diameter in the whole system, also the melting temperature of the fibers was similar to the melting temperature of P(VDF-TrFE) resin powder (140.8 °C).

When it comes to the ferroelectric to paraelectric (FE–PE) transition (also known as the Curie transition), we observed a gradual transition from multiple peaks to one uniform peak when the mean diameter increased. The total heat of FE–PE transition (ΔH_C_) vs the fiber mean diameter is shown in Fig. 5(b). The data show that ΔH_C_ remains almost unchanged until the mean diameter of the fibers reaches 416 nm. At higher fiber diameter, ΔH_C_ showed an increase with the fiber diameter, which could be attributed to the perfection of the ferroelectric domains at higher fiber diameters (>450 nm). This demonstrates that low molecular weight P(VDF-TrFE) with high concentrations could promote better ferroelectric domains.

We also studied the changes of fiber morphology by tuning the flow rate, focusing on the 36 w/v% P(VDF-TrFE) solution for which the flow rate varied from 1 to 3 ml/h. The results presented in Figs. S2 and S3(a) in the supplementary material indicate that increasing flow rate with all other parameters held fixed efficiently increases the mean fiber diameter. In particular, the maximum mean fiber was achieved at flow rate 3 ml/hm reaching 1.175 *μ*m, which is double the mean diameter achieved at a flow rate of 1 ml/h. For this larger sample, the estimated β phase at 1287 cm^−1^ was maintained at 77% in the nanofibers prepared with 36 w/v% solution, while the flow rate changed from 1 to 3 ml/h, as shown in Fig. S3(b) in the supplementary material. Thus, increasing the flow rate does not affect the domination of β phase, but it does significantly increase the mean fiber diameter.

## IV. CONCLUSIONS

Here, we investigated the relationship of morphology, viscosity, the *Be* of solutions of low molecular weight P(VDF-TrFE) (70/30) in mixed solvent (DMF/acetone 5/4) with different concentrations. The estimated crystallinity from XRD results of electrospun P(VDF-TrFE) nanofiber confirmed that a high crystallinity level (67.2%) was reached in electrospun P(VDF-TrFE) (70/30) fibers with a mean diameter 416 nm, which was prepared with a relatively low molecular weight polymer. Our FTIR study further revealed that all-trans conformation of 79% persisted as the dominant in chain conformation across all electrospun nanofibrous webs with mean diameter spanning 285 to 592 nm. This confirmed that the electrospinning of low molecular weight P(VDF-TrFE) (70/30) not only provides molecular orientation during electrospinning process but also enhances the crystallinity within the constituent nanofibers, without any annealing treatment. Consistent with FTIR and XRD studies, the DSC results indicated that the ferroelectric domains became less defective and increased in the size for samples featuring larger mean fiber diameter, consistent with the higher crystallinity, and explained on the basis of the slowing evaporation rate for higher concentration (larger diameter jet) solutions.

## SUPPLEMENTARY MATERIALS

See the supplementary material for the calculation to obtain the Berry number, the effect of the flow rate on the fiber diameter size, and the estimated all-trans fraction with the changes in the flow rate.

## Data Availability

The data that support the findings of this are available from the corresponding authors upon reasonable request.
